# Multi-omic derived cell-type specific Alzheimer disease polygenic risk scores

**DOI:** 10.1016/j.neurobiolaging.2025.07.009

**Published:** 2025-07-16

**Authors:** Nicholas O’Neill, Nuzulul Kurniansyah, Congcong Zhu, Oluwatosin A. Olayinka, Richard Mayeux, Jonathan L. Haines, Margaret A. Pericak-Vance, Li-San Wang, Gerard D. Schellenberg, Lindsay A. Farrer, Xiaoling Zhang

**Affiliations:** a Bioinformatics Program, Boston University, Boston, MA, USA; b Departments of Medicine (Section of Biomedical Genetics), Boston University Chobanian & Avedisian School of Medicine, Boston, MA, USA; c Departments of Neurology, Boston University Chobanian & Avedisian School of Medicine, Boston, MA, USA; d Departments of Ophthalmology, Boston University Chobanian & Avedisian School of Medicine, Boston, MA, USA; e Department of Neurology, Columbia University School of Medicine, New York, NY, USA; f Cleveland Institute for Computational Biology, Department of Population and Quantitative Health Sciences, Case Western Reserve University, Cleveland, OH, USA; g John P. Hussman Institute for Human Genomics, Miller School of Medicine, Miami, FL, USA; h Department of Pathology and Laboratory Medicine, University of Pennsylvania Perelman School of Medicine, Philadelphia, PA, USA; i Departments of Biostatistics, Boston University School of Public Health, Boston, MA, USA; j Departments of Epidemiology, Boston University School of Public Health, Boston, MA, USA

**Keywords:** Alzheimer disease, Polygenic risk scores, snATAC-seq, Amyloid pathology, Somatostatin, neuropeptide Y

## Abstract

Alzheimer disease (AD) polygenic risk scores (ADPRS) built from cell-type (ct) specific genetic variants can be used to infer cell-type contributions to AD. We derived two ct-ADPRSs using variants near single-nuclei RNA-seq (snRNA) derived cell-type specific genes or on single-nuclei ATAC-seq (snATAC) derived cell-type specific accessible chromatin regions. We generated a multi-omic ct-ADPRS for eight neuron subtypes using both single-nuclei datasets. SnATAC-derived ct-ADPRSs demonstrated considerably lower correlations among cell types (average r = 0.071) than snRNA-derived ct-ADPRSs (average r = 0.19), indicating their heightened cell-type specificity. The association of these ct-ADPRSs with AD endophenotypes was evaluated using logistic and linear regression models. Tau tangle burden was associated with astrocyte (AST) ct-ADPRS derived from snATAC (β=0.82, FDR=0.0013) and snRNA (β=0.60, FDR=0.045) as well as microglia (MIC) ct-ADPRS from both (snATAC: β=0.75, FDR=0.0047) (snRNA: β=0.63, FDR=0.028). AST ct-ADPRS was significantly associated with Mini-Mental State Examination score only when derived from snATAC data (β=−0.82, FDR=0.011). SST expressing GABAergic neuron ADPRS was strongly associated ct-ADPRS with neuritic plaque burden (β=0.087 FDR=0.0014) and the only neuron subtype ct-ADPRS significantly associated with AD endophenotypes. We investigated 1954 SNPs contributing to this ct-ADPRS and found the strongest association with variants upstream of the neuropeptide Y gene, *NPY*, particularly rs3940268 (β=−0.13, P = 8.2×10^−5^). This association is significant even after adjusting for diffuse plaque (β=−0.12, P = 1.5×10^−4^) or neurofibrillary tangle burden (β=−0.08 P = 3.9×10^−3^). *NPY* was expressed in a small subset of neurons, and these findings suggest its strong impact on the association of SST+ GABAergic neurons with early AD pathology.

## 1. Introduction

Alzheimer’s disease (AD) is a complex dementing illness affecting millions worldwide and growing in prevalence as our population ages (Li et al., 2022). The progression of AD consists of increasing amyloid-β (Aβ) accumulation followed by glial responses, neurofibrillary tangle (NFT) formation, neuronal loss, and cognitive decline (Selkoe and Hardy, 2016). In addition, aging participants exhibit a wide range of pathological and cognitive states, including tau-positivity without any Aβ pathology (Yoon et al., 2022) and extensive NFTs but no evidence of cognitive decline, indicative of cognitive reserve (Holtzman et al., 2011). Resilience to AD pathology has been linked to neuronal DNA damage response (Mathys et al., 2023), lack of comorbid pathologies (Aiello Bowles et al., 2019), and the maintenance of neuron populations (O’Neill et al., 2024; Perez-Nievas et al., 2013).

The heritability of AD is high, estimated to be 60–70 % (Gatz et al., 2006). Genome-wide association studies (GWAS) have identified ~100 loci associated with AD across multiple ancestries (Bellenguez et al., 2022; Kang et al., 2024; Kunkle et al., 2021; Sherva et al., 2023), and the *APOE* ε4 allele is the largest genetic risk factor for late-onset AD (Bellenguez et al., 2022; Yan et al., 2021), disproportionally important to microglia (MIC) (Efthymiou and Goate, 2017). By aggregating the effects of known AD-risk genetic variants, AD polygenic risk scores (ADPRSs) can be calculated (Leonenko et al., 2021; Tan et al., 2019), which may be used to differentiate high and low-risk individuals (Leonenko et al., 2021) for clinical trials. ADPRS is also associated with Aβ and NFT severity, cognitive decline, and common comorbid pathologies such as Lewy body disease and cerebrovascular disease (Leonenko et al., 2021; Tan et al., 2019), and can be integrated in studies focused on particular biological pathways.

Recent large-scale single-nuclei RNA sequencing (snRNA) studies have enhanced our understanding of AD progression and the role of cell types (Grubman and Polo, 2019; Lau et al., 2020; Mathys et al., 2023, 2019). MIC states, characterized by snRNA, are implicated in the clearance of Aβ, and inflammatory states are associated with the spread of tau (Gao et al., 2023; Sun et al., 2023). The role of astrocytes (AST) in AD is less well characterized, although a subtype of activated AST has been found to associate with disease progression and localize to amyloid plaques (Habib et al., 2020). Studies have also identified neuron subtypes whose loss is strongly related to cognitive decline, such as Parvalbumin (*PVALB)* (Ali et al., 2019; Consens et al., 2022; Gabitto et al., 2023; O’Neill et al., 2024) and Somatostatin (SST) positive GABAergic (GAB) (Almeida, 2024; Cain et al., 2023; Consens et al., 2022; Gabitto et al., 2023; Mathys et al., 2023; Waller et al., 2020). Elucidating associations of specific cell types and subtypes with AD endophenotypes will likely deepen our understanding of AD progression and may better explain disparities between pathological and cognitive states.

Recently, Yang et al. combined the cell-type (ct) specificity of snRNA with GWAS summary statistics of AD to calculate cell-type specific ADPRS (ct-ADPRS) scores by aggregating single nucleotide polymorphisms (SNPs) near genes expressed specifically in each primary cell type in the brain (Yang et al., 2023). Because SNPs are selected solely on their implied cell-type effect specificity, a ct-ADPRS best summarizes the genetic influences of a cell-type’s involvement in AD-related pathology. This idea is supported by findings that an AST-ADPRS was significantly associated with the severity of tau and Aβ pathology and a MIC-ADPRS was significantly associated with cognitive decline, tau and neuritic plaque burden. Approximately 16 % of the identified cell-type-specific genes were shared between two or more cell types, particularly MIC and ODC (Yang et al., 2023).

In this study, we used a single cell level assay for transposase-accessible chromatin (ATAC) data and single-cell RNA sequencing (snRNA) data generated from brain tissue donated by participants of the Religious Orders Study and the Rush Memory and Aging Project (ROS-MAP) to calculate ct-ADPRSs by identifying SNPs on cell-type specific snATAC open chromatin regions (peaks), which more precisely capture differences between cell types than SNPs near cell-type specific snRNA genes. We investigated the association of ct-ADPRSs, including a neuron subtype multi-omic ct-ADPRS, with AD-related neuropathological and cognitive traits.

## 2. Materials and methods

### 2.1. Participants, diagnostic procedures, and data processing

Cognitive, neuropathological, and whole genome sequencing data for 1111 ROSMAP participants (median age = 89, 65.7 % women) of European ancestry were extracted from ADSP release 3 (R3) (https://adsp.niagads.org/data/data-summary/), a dataset comprised of 16,905 participants with genotypes jointly called by the Genome Center for Alzheimer’s Disease (GCAD) (Leung et al., 2023). Participants were classified as control with no cognitive impairment (n = 351), mild cognitive impairment (MCI, n = 258), or AD (n = 407) according to NIA-Reagan Criteria (Schneider et al., 2007). Participants with non-AD causes of cognitive impairment were excluded. Baseline characteristics of ROSMAP participants are shown in Supplementary Table 1. Genetic variants with minor allele count (MAC) < 5, not in Hardy-Weinberg equilibrium (HWE, P < 1.0×10^−6^), or missing in > 10 % of participants were excluded. Principal components (PCs) of ancestry were generated using PLINK version 1.90 (Chang et al., 2015), using a set of variants with minor allele frequency (MAF) > 0.05, in HWE and not in linkage disequilibrium (LD, R^2^*<*0.25), and missingness rates < 1 % for samples and variants.

### 2.2. Genome-wide association analyses

We generated AD GWAS summary statistics using the Alzheimer’s Disease Genetics Consortium (ADGC) European ancestry cohorts without ROSMAP (Bellenguez et al., 2022; Kunkle et al., 2019) using SAIGE (Version 1.1.9) (Zhou et al., 2018). After excluding cohorts with fewer than five participants in AD case or control groups, 33 cohorts containing 16,230 AD cases and 17,643 controls remained for subsequent analyses (Supplementary Table 2). SNP genotypes were imputed using the NHLBI TOPMed v5 reference panel (Taliun et al., 2021). Variants with MAF < 0.01 and imputation quality score (R^2^) < 0.8, and variants with MAF > 0.01 and R^2^ < 0.4 were excluded. The association of AD with each SNP was evaluated in each cohort using logistic regression models including covariates for age, sex, and the first five PCs. Result for all cohorts were combined by meta-analysis using a fixed-effects inverse variance approach implemented in METAL (Willer et al., 2010).

### 2.3. Cell-type specific gene and peak sets

**Fig. 1.**
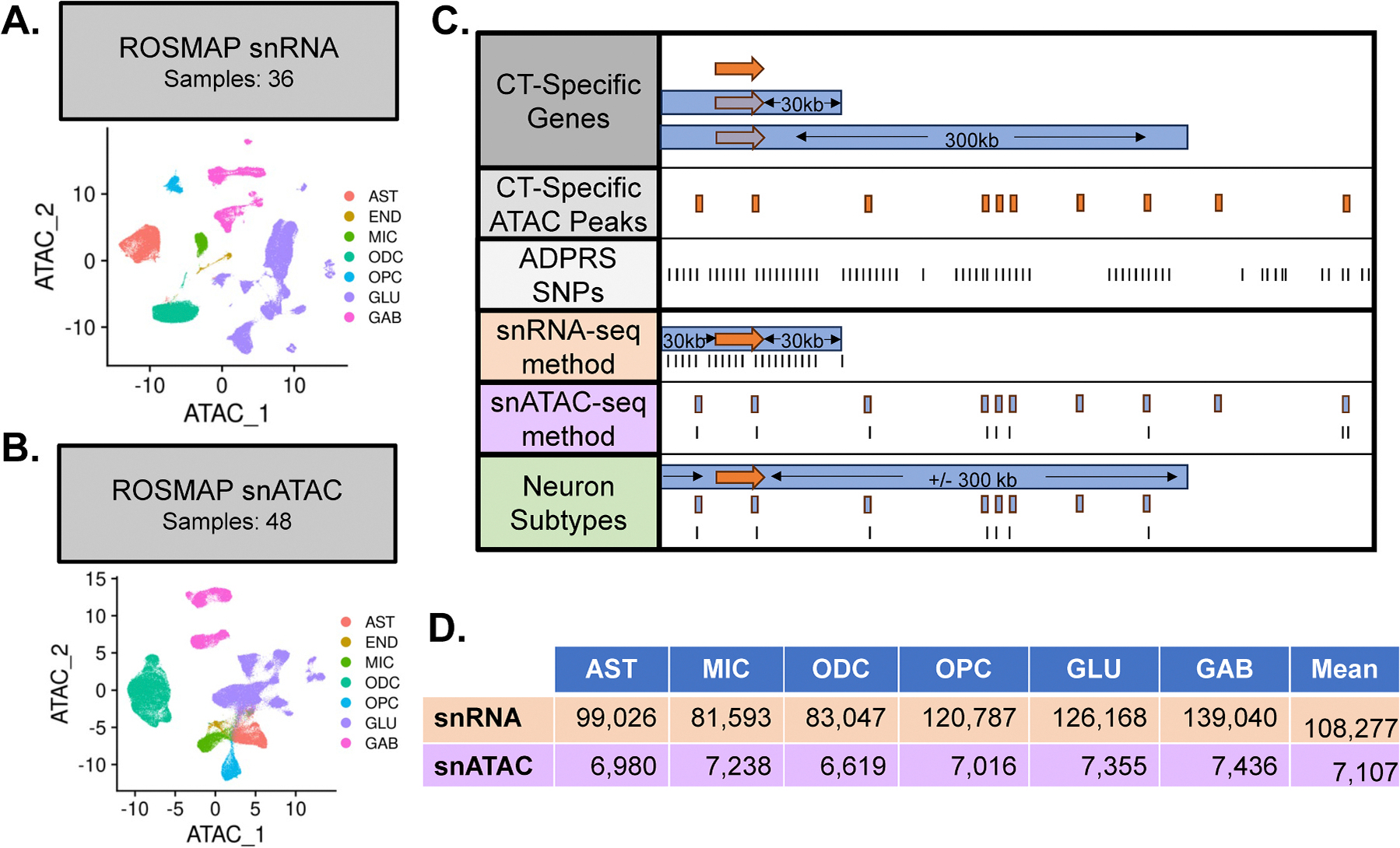
Overview of study design. **A**). Uniform manifold approximation and projection (UMAP) of the seven primary cell-types in the ROSMAP snRNA data used to select cell-type specific genes. MIC = microglia; ODC = oligodendrocytes; AST = astrocytes; OPC = oligodendrocyte precursors; END = endothelial cells; GLU = glutamatergic neurons; GAB = GABAergic neurons. **B**). UMAP of seven primary cell-types in the ROSMAP snATAC data used to select cell-type specific open chromatin regions, or “peaks”. **C**). Cartoon demonstrating the SNP selection for the three ct-ADPRS methods. 1st row: Cell-type specific genes are selected using snRNA data. 2nd row: Cell-type specific peaks are selected using snATAC data. 3rd row: SNPs included in our AD summary statistics derived from ADGC European ancestry participants with ROSMAP participants removed. 4th row: For snRNA ct-ADPRS we select SNPs that are on or within 30 kb, upstream or downstream, of cell-type specific genes. 5th row: SnATAC-derived ct-ADPRSs use SNPs that intersect with cell-type specific snATAC peaks. 6th row: Neuron subtype ct-ADPRSs select SNPs that intersect neuron subtype specific snATAC peaks and are on or within 300 kb, upstream or downstream, of neuron specific genes. **D**). Table showing the number of SNPs included in both basic ct-ADPRSs for each of the six cell-types considered. The mean SNP count for each method is shown in the final column.

We identified cell-type-specific gene sets using publicly available snRNA data generated from brain tissue donated by ROSMAP Study participants (Fujita et al., 2024) (Fig. 1a) using an approach similar to that applied in a previous study (Yang et al., 2023). Specifically, we calculated the average expression of genes among 36 controls, defined as having possible or no neuritic plaques, a Braak score of ≤ 2, no evidence of cognitive decline, and an MMSE score > 23, for seven primary cell types: astrocytes (AST), microglia (MIC), oligodendrocytes (ODC), oligodendrocyte precursors (OPC), endothelial cells, and excitatory (GLU) and inhibitory (GAB) neurons using Seurat (Version 5.1.0). Gene specificity to each cell type ($S_{gc}$) was calculated using the following formula:

$$S_{gc}=\frac{E_{gc}}{\sum_{c=1}^{C}\;E_{gc}}$$

where $E_{gc}$ is the average expression for gene (g) in cell-type (c). We included genes expressed in at least 10 % of cells in at least one cell type (n = 14,000) and selected the top 10 % most specific genes for each cell type from that list (n = 1400).

Employing the same approach, we identified cell-type specific snATAC sites for the same seven primary cell types using snATAC peak score matrices and limited to nuclei with transcription start site (TSS) enrichment greater than one that were previously generated from ROSMAP participant brain tissue (Xiong et al., 2023) (Fig. 1b). We calculated the term-frequency inverse-document-frequency for the 300, 000 snATAC 500 bp peaks using Signac (Version 1.13.0) and the average activity score ($A_{pc}$) per peak (p) and cell-type (c) for nuclei derived from the 48 pathologically diagnosed controls and with TSS enrichment greater than 1. Next, we calculated the cell-type specificity scores ($S_{pc}$) as follows:

$$S_{pc}=\frac{A_{pc}}{\sum_{c=1}^{C}\;\;A_{pc}}$$

We selected the top 10 % of peaks for each primary cell type (n = 29,407) as cell-type specific ATAC peaks.

We used Signac functions “FindTransferAnchors” and “TransferData” (Stuart et al., 2019) to transfer neuron subtype identities we previously characterized (O’Neill et al., 2024) in a ROSMAP snRNA dataset (Mathys et al., 2019) to the snATAC neurons using gene score matrices (Xiong et al., 2023). Neuron subtypes include GAB neurons expressing SST, PVALB, VIP, and LAMP5 and GLU neurons expressing THEMIS, SEMA3E, RORB, and LAMP5. The average activity score (A_pc_) per peak and neuron subtype were calculated using these data. To identify GAB neuron subtype-specific peaks we selected the top 20 percent of cell-type specific peaks for GABAergic neuron (GAB, n = 58, 815) and then calculated the cell-type specificity scores (S_pc_) for the four GAB subtypes. We selected the top 20 percent most specific peaks on this subset (n = 11,762) to represent each subtype. The same procedure was used to identify GLU neuron subtype-specific peaks. The snRNA-seq data and neuron subtypes were also used to generate violin plots and UMAPs of gene expression.

### 2.4. Cell-type-specific ADPRS calculation

We calculated a ct-ADPRS using three approaches (Fig. 1c). First, for six primary cell-types we created a snRNA gene-based ct-ADPRS using SNPs located within 30 kb of cell-type specific genes in a manner similar to the procedure applied by Yang et al. (Yang et al., 2023). Second, we considered SNPs on cell-type specific ATAC peaks as a snATAC derived ct-ADPRS for primary cell-types. Endothelial cells were excluded from further analysis due to their low representation in the snRNA and snATAC data (≤1.35 %) (Supplementary Table 3). Third, for neuron subtype-specific ct-ADPRSs ranges we use a single, multi-omic approach. We limited subtype-specific peaks to those within 300 kb of a GAB or GLU specific gene, defined more leniently as having S_gc_ in the top 20 percent. SNPs within 1MB of the *APOE* coding region were removed from each ct-ADPRS range.

The posterior effect size of each SNP was estimated by effect size shrinkage using default parameters of PRS-CS software (Ge et al., 2019) and the 1000 Genome Project whole genome sequence data for Europeans (1000 G EUR) provided by PRS-CS (Auton et al., 2015) as an LD reference panel. We calculated ct-ADPRS scores for ROSMAP participants using PRSice (version 2.3.3), limited to the specific ranges for the three cell types defined in previous steps, and the ADGC GWAS summary statistics. We also generated “Genome” ADPRS scores without restricting loci to cell-type-specific regions that were derived from all 990,428 SNPs. The overlap between SNPs used for snRNA and snATAC ct-ADPRS methods was visualized in an UpSet plot generated by the ComplexHeatmap (2.14.0) R package.

### 2.5. Association of ct-ADPRSs with AD clinicopathological traits

The ct-ADPRS scores were standardized by subtracting the sample mean score and dividing the difference by the standard deviation to yield a normal distribution. Association of the standardized ct-ADPRS with multiple outcomes (pathological AD diagnosis, MMSE score, PHF Tau tangle density, NFT burden, overall Aβ burden, diffuse plaque burden, and neuritic plaque burden) was evaluated using logistic or linear regression models including terms for age at death, sex, the first three PCs, and dosages of the *APOE* ε2 and ε4 alleles. Years of education were included in models examining associations with MMSE score. The False discovery rate (FDR) for the primary cell-type analysis was calculated by multiplying p-values by six, the number of primary cell types tested. FDR for the neuron subtype analysis was calculated from p-values by adjusting for the eight subtypes tested. To reach an FDR of 0.05, the p-value must be 0.00833 in the primary analysis or 0.00625 in the subtype analysis.

### 2.6. SST positive GABAergic neuron ct-ADPRS investigation

We tested the association of the 1954 SNPs contributing to the SST+ GABAergic neuron ct-ADPRS with neuritic plaque and Aβ burden using linear regression adjusted for age at death, sex, the first three PCs, and dosages of the *APOE* ε2 and ε4 alleles. We similarly tested the association between AD clinicopathological traits and SNPs within 300 kb of rs3940268 or rs10941583, the SST+ GABAergic neuron SNPs most strongly associated with neuritic plaque and Aβ burden, respectively. rs3940268 is upstream of the gene *NPY,* and rs10941583 is in an intronic region of *GHR*. For the 390 participants with snRNA-seq and WGS data, we tested the association between variants around rs3940268 and the expression of *NPY* using a linear model and adjusting for the same covariates. We further evaluated the normalized expression patterns of *NPY* and *GHR* in the 36 controls with snRNA-seq data using Seurat (Version 5.1.0).

## 3. Results

### 3.1. Cell-type specificity and function of ct-ADPRS strategies

**Fig. 2.**
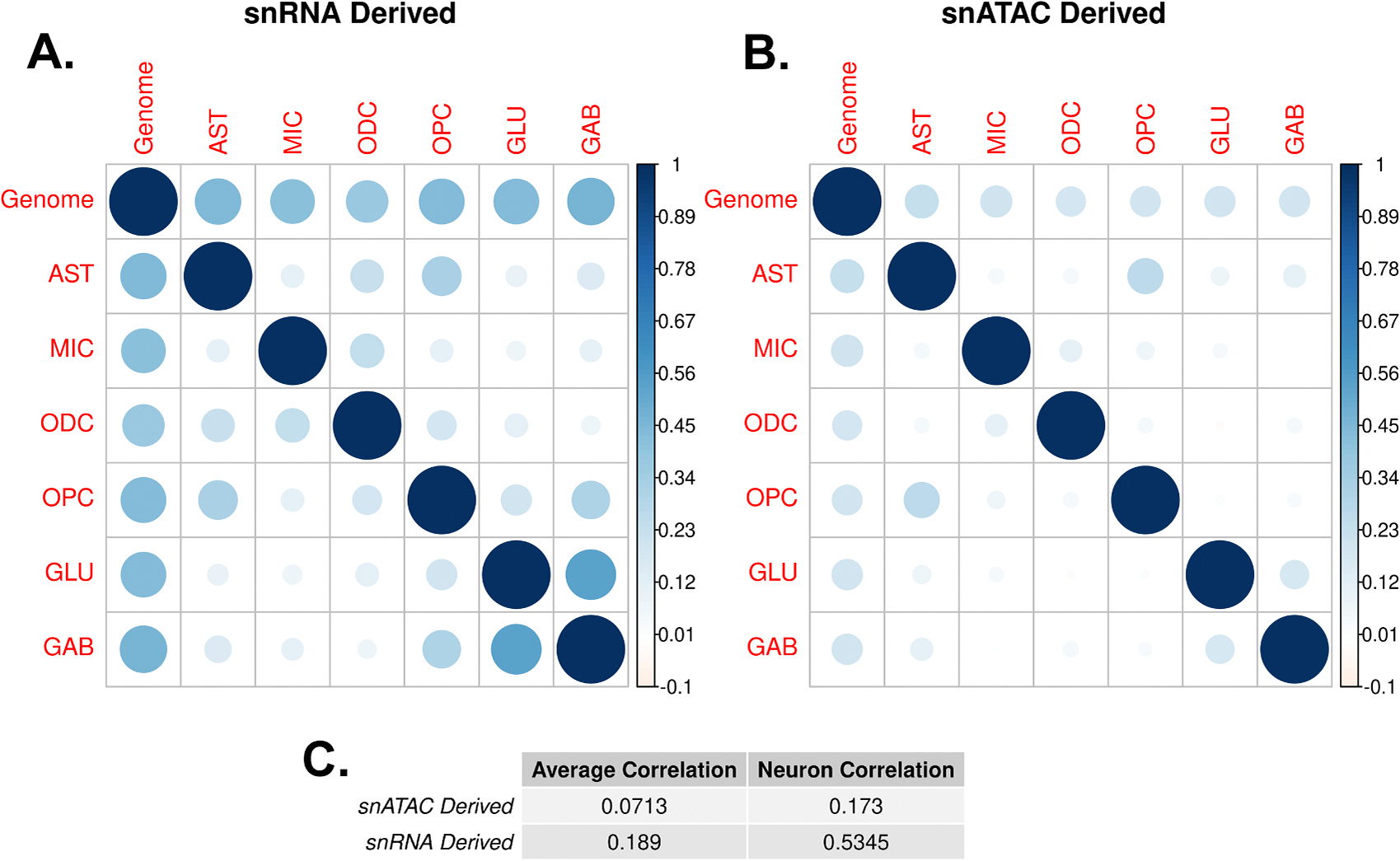
Correlation between ct-ADPRSs. **A-B**). Correlation plots of ct-ADPRS and overall ADPRSs for each PRS calculation strategies. Blue circles represent correlation ‘r’ values between two cell-types’ ADPRSs. The first row and column of each correlation plot is the overall ‘Genome’ ADPRS generated by using all SNPs, regardless of cell-type specificity. **C**). Average correlation ‘r’ values between each cell-type, or between glutamatergic and GABAergic neurons, for each PRS calculation strategy.

Cell-type-specific variants were identified from snRNA (Fig. 1a) and snATAC (Fig. 1b) profiles, which were used to generate snRNA and snATAC ct-ADPRSs (Fig. 1c). Gene and peak specificity distributions and the specificity thresholds are shown in Supplementary Fig. 1A–B. The mean SNP count for each ct-ADPRS method differed greatly. SnRNA-derived ct-ADPRSs averaged 108,277 SNPs and snATAC-derived ct-ADPRSs averaged 7107 SNPs, or 6.6 % of the snRNA-derived count (Fig. 1d). In the aggregated cell types, an average of 33.6 % of SNPs included in the snATAC-derived ct-ADPRS were included in the equivalent snRNA-derived ct-ADPRS. Among each of the six primary cell types examined, variant sets derived from snATAC are more cell-type specific than those from snRNA, with 88.2 % of variants being unique to one cell-type compared to only 57.5 % of snRNA derived variants (Supplementary Fig. 2). Interestingly, MIC has the fewest associated variants in snRNA-derived ct-ADPRS but the largest variant count among glia in the snATAC set. In addition, whereas the average correlation among paired comparisons of standardized ct-ADPRS derived from snRNA (r = 0.189) is moderately greater than the one for snATAC (r = 0.071), the difference is particularly pronounced for the correlation between excitatory and inhibitory neurons derived from snRNA (r = 0.534) compared to that derived from snATAC (r = 0.173) (Fig. 2). The specificity thresholds for peaks are higher than those for peaks in every cell type except for endothelial cells (Supplementary Fig. 1C). Correlations among neuronal subtype ct-ADPRS and overall ADPRS are shown in Supplementary Fig. 3 and the number of SNPs included in the calculation of each subtype ct-ADPRS is provided in Supplementary Table 4.

### 3.2. Association between ct-ADPRSs with AD clinical and pathological traits

**Fig. 3.**
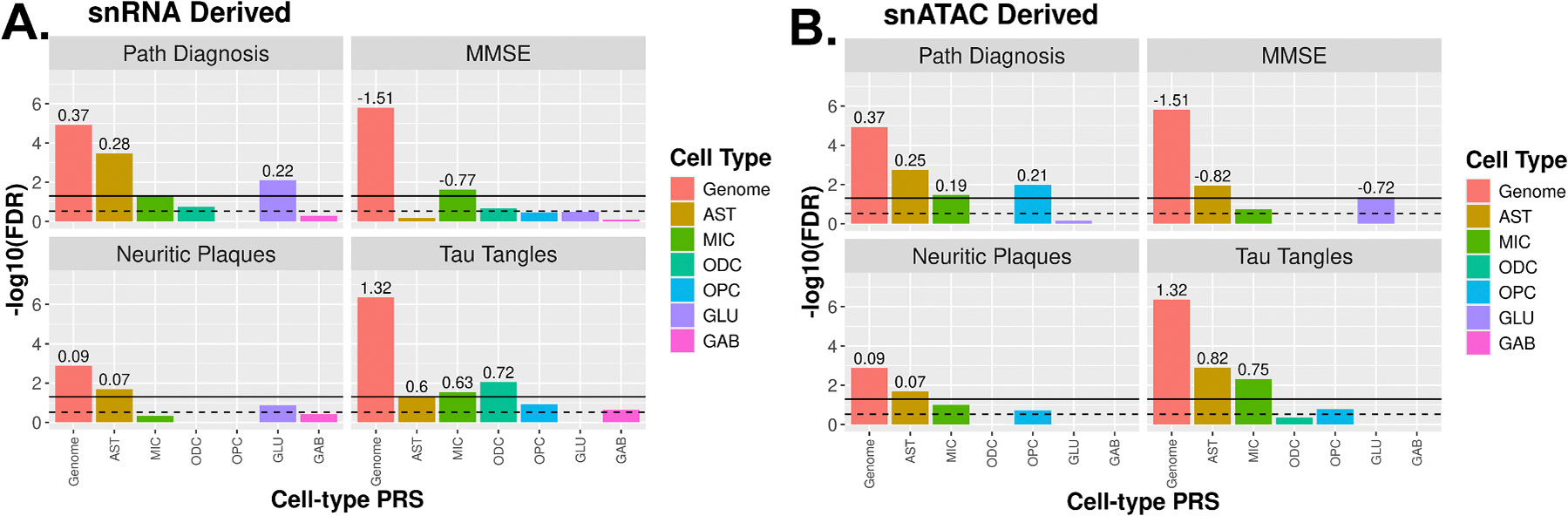
ct-ADPRS association with AD endophenotypes. For each ct-ADPRS, **A**). snRNA derived and **B**). snATAC derived, we show logistic model results for pathological AD diagnosis (niareagan 0–1 vs 2–3) and linear model results for MMSE score, neuritic plaque burden quantified by microscopy, and immunohistochemistry-derived paired helical filament tau tangle density. Models are adjusted for age, sex, APOE e2 and e4 dosage, and three genetic principal components. MMSE models also include a covariate for years of education. Bars represent the −log10(FDR) for each of the six cell-types considered as well as ‘Genome’ which represents an ADPRS generated using all AD SNPs. The dotted line represents a p-value of 0.05 while the black lines represent an FDR of 0.05. Effect size is shown above each bar for each significant ADPRS.

**Fig. 4.**
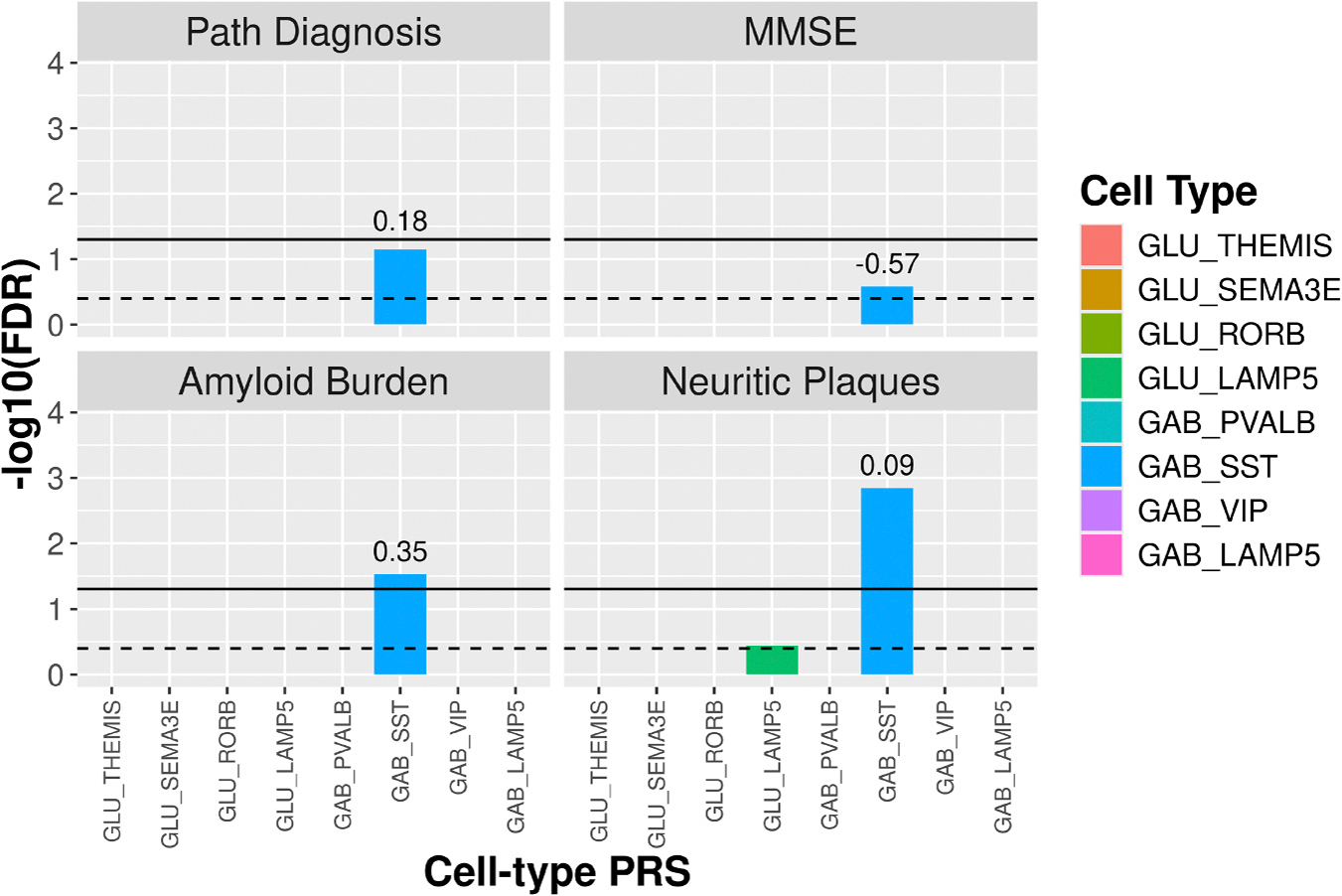
Multi-omic ct-ADPRS for neuron subtypes. We calculate a ct-ADPRS for each of eight neuron subtypes, four GABAergic and four glutamatergic. For each subtype we show logistic model results for pathological AD diagnosis (niareagan 0–1 vs 2–3) and linear model results for MMSE score, Aβ burden quantified by immunohistochemistry, and neuritic plaque burden quantified by microscopy. Models are adjusted for age, sex, APOE e2 and e4 dosage, and three genetic principal components. MMSE models also include a covariate for years of education. Bars represent the −log10(FDR) for each of the eight neuron subtypes. The dotted line represents a p-value of 0.05 while the black lines represent an FDR of 0.05. Effect size is shown above each bar for each significant ADPRS.

MMSE score was significantly associated with the snATAC AST (β=−0.82, FDR=0.011) and GLU (β=−0.72, FDR=0.044) ct-ADPRSs (Fig. 3, Supplementary Table 5, Supplementary Fig. 4), whereas MMSE score was significantly associated with the MIC ct-ADPRS calculated by the snRNA method only (β=−0.77, FDR=0.024, Supplementary Table 6). Tangle burden was significantly associated with ct-ADPRSs calculated by either method for AST (snRNA, β=0.60, FDR=0.045; snATAC, β=0.82, FDR=0.0013) and MIC (snRNA, β=0.63, FDR=0.028; snATAC, β=0.75, FDR=0.0047). The snRNA-derived ODC ct-ADPRS was also significantly associated with tangle burden (β=0.72, FDR=0.0089). Pathological AD diagnosis was significantly associated with AST ct-ADPRs calculated by both snRNA (β=0.28, FDR=0.00033) and snATAC (β=0.25, FDR=0.0018) approaches. Only two significant associations with the multi-omic neuron subtype ct-ADPRS were observed including SST+ GABAergic neuron ct-ADPRS with Aβ burden (β=0.35, FDR=0.030) and neuritic plaque burden (β=0.087, FDR=0. 0014) (Fig. 4, Supplementary Table 7, Supplementary Fig. 5).

### 3.3. SST positive GABAergic neuron ct-ADPRS investigation

**Fig. 5.**
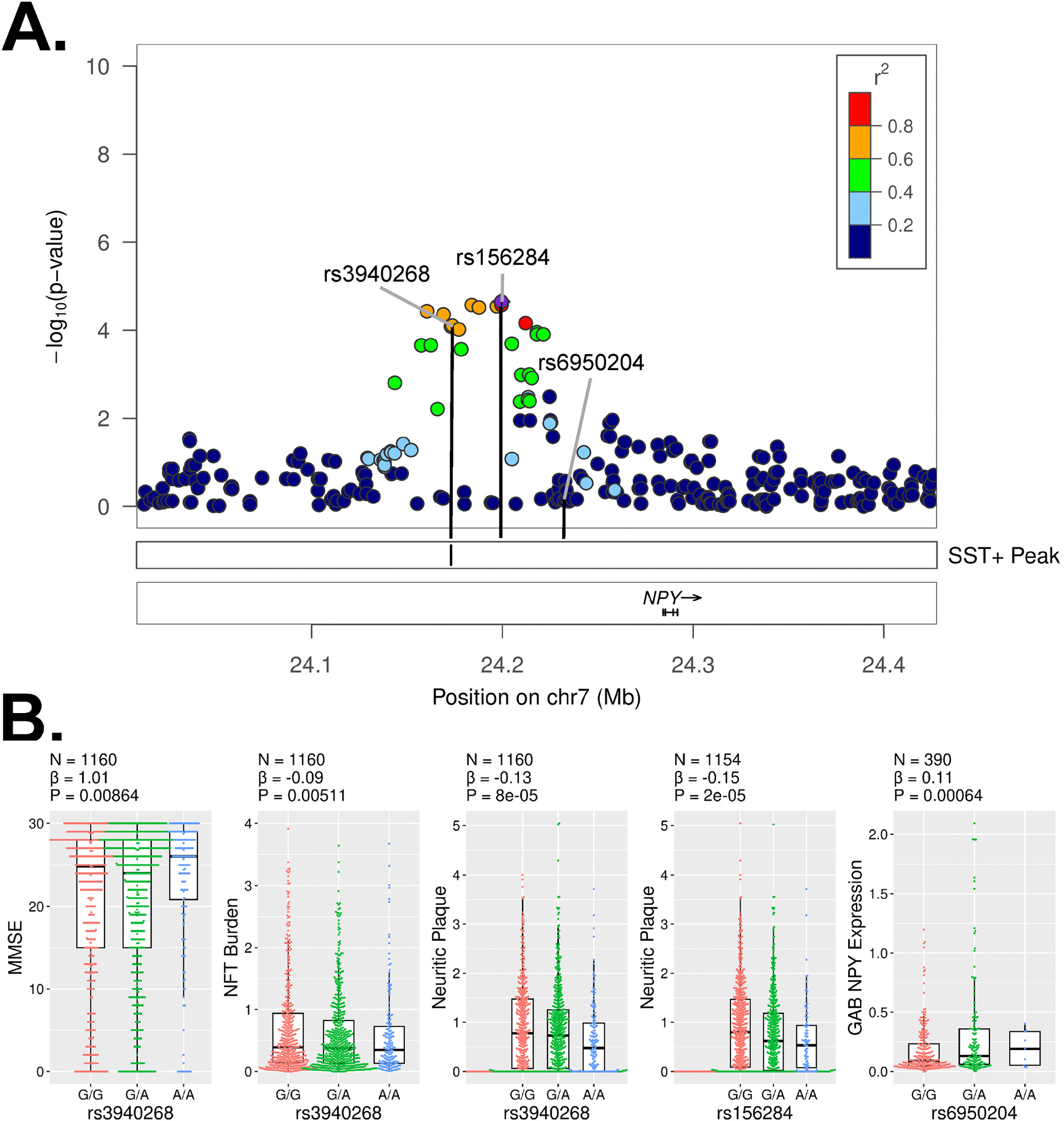
SNPs of interest upstream of NPY. **A**). Locus zoom plot showing the association between neuritic plaque burden with variants near the *NPY* gene. The X-axis signifies the SNP position and the significance of association is shown by the −log_10_(p-value) on the Y-axis. The location of the SST+ GABAergic neuron specific ATAC peak is labeled. **B**). Box plots relating selected SNPs with participant traits or NPY expression. N represents the number of participants in the linear model comparison and β and P values shown represent the association between the labeled SNP and the trait, adjusting for age, sex, PMI, APOE2–4 status, and three genetic prin-cipal components.

To better understand the association of *SST*-positive GABAergic neurons with amyloid pathology, we examined the locations of the 1954 SNPs contributing to its ct-ADPRS. A near study-wide significant association (P < 2.26×10^−5^) for neuritic plaque burden was observed with rs3940268 (β=−0.13, P = 8.2×10^−5^, Fig. 5, Supplementary Table 8) which is located 111 kb upstream of the nearest gene, NPY. This association is still significant after adjusting for diffuse plaque burden (β=−0.12, P = 1.5×10^−4^) or NFT burden (β=−0.08, P = 3.9×10^−3^). Rs3940268 is also associated with MMSE score (β=1.01, P = 8.6×10^−3^) and NFT burden (β=−0.09, P = 5.1×10^−3^, Fig. 5), though the association with NFT burden is not significant after adjusting for neuritic plaque burden (β=−0.02, P = 0.42). Examination of nearby SNPs, including those not included in the calculation of the SST+ ct-ADPRS, revealed nine SNPs slightly more significantly associated with neuritic plaque burden, including rs156284 (β=−0.15, P = 2.2×10^−5^, Fig. 5, Supplementary Table 9). Further analysis showed that an adjacent SNP, rs6950204, is significantly associated with *NPY* expression in GABAergic neurons (β=0.11, P = 6.4×10^−4^, Fig. 5). *NPY* is most strongly expressed in a small subset of SST+ GABAergic neurons in the brain (Supplementary Fig. 6). This subset of SST+ GAB neurons also expresses CHODL, TACR1, and NOS1 (Supplementary Fig. 7).

The *SST* ct-ADPRS variant most significantly associated with Aβ burden is *GHR* intronic SNP rs10941583 (β=0.7, P = 3.16×10^−4^, Supplementary Fig. 8). Six other nearby GHR SNPs not included in the SST+ ct-ADPRS calculation were more significantly associated including rs10038285 (β=0.75, P = 0.8.72×10^−5^, Supplementary Fig. 8, Supplementary Table 10).

## 4. Discussion

We calculated cell-type specific PRSs for AD using gene expression data derived from snRNA-seq and snATAC-seq data and genome-wide SNP data generated from brain tissue donated by ROSMAP Study participants. We identified significant associations of measures of AD pathology severity and MMSE score with AST ct-ADPRSs that were derived from snATAC-seq data using an average of 6.6 % of the SNPs included in the calculation of ct-ADPRSs derived from snRNA-seq data. We also found that *SST*+ GAB ADPRSs are strongly associated with Aβ level and neuritic plaque burden with the most associated SNPs at the *NPY* and *GHR* loci.

The value of a ct-PRS for investigating the role of cell types in AD is directly related to its cell-type specificity. Inclusion of non-cell-type-specific variants will obscure the distinction between a ct-ADPRS and the general ADPRS. Highlighting this difficulty, Yang et al. compensated for SNPs shared between ct-ADPRSs by conducting ct-ADPRS association analyses using MIC ct-ADPRS as a covariate or excluding genes shared between ADPRSs (Yang et al., 2023). Indeed, using a different single-nuclei expression reference and set of GWAS summary statistics, we found that the ODC ct-ADPRS derived only from snRNA-seq data was significantly associated with tau tangle burden. Our analyses, incorporating ct-ADPRSs calculated from snATAC-seq data, minimized the correlations of ct-ADPRSs among cell types and the general ADPRS and identified significant associations of glial cells with AD-related clinical and neuropathological traits.

Our analyses of ct-ADPRSs calculated using snATAC cell-type data identified novel associations that were weaker or absent from analyses of ct-ADPRSs derived from snRNA-seq data. This improvement is consistent with a coronary artery disease study that found improved cell-type specific PRS performance upon consideration of snATAC peaks in variant selection (Örd et al., 2023). The MIC ct-ADPRS calculated from either snRNA-seq or snATAC-seq data was associated with pathological AD diagnosis and tau tangle burden, but the association of the AST ct-ADPRS with NFT burden was significant only for scores derived from snATAC-seq data. Our association finding for the AST ct-ADPRS and tau pathology is supported by studies showing that reactive AST is a driver of tau pathology in humans (Bellaver et al., 2023) and mice (Bellenguez et al., 2022; Litvinchuk et al., 2018). While both MIC and AST have been linked to AD pathology (Gao et al., 2023; Habib et al., 2020; Leng and Edison, 2021; Rodriguez-Vieitez et al., 2015) and thus may be associated with consequential cognitive decline (Selkoe and Hardy, 2016), AST biomarkers have been associated with AD severity, and cognitive decline at different stages of the disease (Holper et al., 2024) which is consistent with our observed association of AST ct-ADPRS with MMSE score. Evidence suggests that MIC activation plays a pivotal role in AD (Gao et al., 2023; Leng and Edison, 2021), but technical limitations make it difficult to fully characterize the cellular subtype with snRNA-seq data (Thrupp et al., 2020). Moreover, we were unable to differentiate MIC subtypes using snATAC-seq data, a limitation that has been previously reported (Sun et al., 2023).

The increased cell-type specificity of snATAC data enabled calculation of neuron-subtype specific ct-ADPRSs. We showed significant associations of *SST*-positive GAB neurons with several measures of amyloid pathology. The strength of the association of the SST-positive GAB neuron ct-ADPRS with neuritic plaque burden was greater than the ADPRS for all other cell-types and similar to the association with the ‘Genome’ ADPRS which was calculated using all SNPs compared to a relatively small number of SNPs for the ct-ADPRS calculation. Prior research demonstrated that *SST*-positive neurons are selectively vulnerable in AD (Almeida, 2024; Cain et al., 2023; Consens et al., 2022; Mathys et al., 2023; Waller et al., 2020) and colocalize with Aβ plaques (Almeida, 2024). The SST protein has been shown to attenuate the cytotoxicity of Aβ species and their interaction with cell membranes (Han et al., 2022).

We observed that among nearly 2000 SNPs contributing to ADPRS for SST-positive GAB neurons, the variant most significantly associated with neuritic plaque burden was an intergenic SNP, rs3940268, whose nearest coding gene is *NPY*. While this analysis is not powered to identify significant SNPs associated with endophenotypes after FDR correction, the p-value would be of suggestive significance in a genome-wide association study. This association was still significant after adjusting for NFT or diffuse plaque burden and appears to be specific to neuritic plaques. Reduced expression of SST and neuropeptide NPY has been correlated with Aβ burden (Ramos et al., 2006; Song et al., 2021) and NPY is downregulated in persons with AD (Tayran et al., 2024). In APP transgenic mouse studies, NPY C-terminal fragment treatment has been shown to ameliorate amyloid pathology (Rose et al., 2009) and cells expressing *NPY* were diminished prior to amyloid accumulation (Mahar et al., 2017). The SST+ GABAergic neuron subtype we showed to express *NPY, TACR1, NOS1*, and *CHODL* was previously identified as long-range-projecting neurons that mediate cerebral blood flow (Ruff et al., 2024). Neurovascular dysfunction is strongly related to AD (Yu et al., 2020) and blood brain barrier dysfunction, which would disrupt Aβ clearance, precedes amyloid accumulation (Sweeney et al., 2018). While it is possible that SST+ GAB loss in AD is related to their selective vulnerability, the association of *SST* expression with Aβ, the colocalization of SST+ neurons with amyloid plaques, and our finding that the SST+ GAB ct-ADPRS is associated with neuritic plaque burden, driven in part by SNPs near *NPY*, indicate a more active role for SST+ neurons in the progression of amyloid pathology. Further research is required to determine if the association of SST+ GAB neurons with neuritic plaques is specific to the subset of neurons expressing *NPY* or is a property of all SST+ GAB neurons. We also observed that several *GHR* intronic variants account for the association of the SST+ GAB ct-ADPRS with Aβ burden. SST+ neurons are responsive to growth hormone and *GHR* ablation in SST+ neurons has been shown to decrease anxiety and fear memory (dos Santos et al., 2023).

Several limitations should be considered in the interpretation of our results. Not all cell-type heterogeneity is captured by snRNA or snATAC, particularly for MIC, and our ct-ADPRSs may have excluded SNPs that influence AD-related pathology in a cell-type specific manner. Second, we evaluated gene expression and chromatin accessibility in samples from cognitively unimpaired individuals and may not have identified cell-type specific genes or peaks found only in AD conditions. Some findings are influenced by our selection criteria for ct-specific SNPs. Third, the distance-based approach we employed for constructing the ct-ADPRS may have omitted relevant SNPs in strong LD with selected variants, potentially leading to some loss of information. However, SNPs that are included and have strong associations are likely to receive higher weights, which helps preserve the predictive power of the PRS. Furthermore, the inclusion of only 300,000 500 bp snATAC peaks greatly limited the area of the genome considered and may not have captured all regions important to each cell type, even if this strategy enriched for regulatory regions. Additionally, although the thresholds for peak and gene specificity lie at the extremes of their cell-type specificity distributions, they are subjectively chosen, and different thresholds could alter the results. Fourth, many of the ct-ADPRS that were significantly associated with AD endophenotypes were not significantly associated with AD status. This may reflect the much greater power to detect associations with continuous traits compared to AD status, a dichotomous outcome which is also subject to competing causes of death. Also, ct-ADPRS associations with AD endophenotypes may reveal important biological mechanisms underlying AD risk and progression without being associated with AD status. Finally, our results are derived primarily from individuals of European ancestry and may not be generalizable to all populations.

In conclusion, this study demonstrated that considering cell-type specific chromatin accessibility and gene expression efficiently selects genomic variants driving cell-type specific contributions to pathological measures of AD. Our findings contribute to a growing body of evidence implicating *NPY* expressing cells in early amyloid pathology and AD. Despite the subpopulation’s rarity it drives SST+ ct-ADPRS association with amyloid pathology and gene expression changes detectable in human studies using bulk RNA-seq. Further research of using large-scale snRNA-seq or spatial transcriptomic data from prodromal and early-stage AD will be essential to understanding the role of SST+ neurons in AD.

## Supplementary Material

Supplementary Tables of Variants

Supplementary Tables of Cohorts

## Data Availability

Data sources are provided within the manuscript.

## Appendix A. Supporting information

Supplementary data associated with this article can be found in the online version at doi:10.1016/j.neurobiolaging.2025.07.009.

## Abbreviations

Aβ: Amyloid-β
AD: Alzheimer disease
ADPRS: AD polygenic risk score
AST: Astrocytes
Ct: Cell-type
Ct-ADPRS: Cell-type specific AD polygenic risk score
GAB: GABAergic inhibitory neurons
GLU: Glutamatergic neurons
MCI: Mild cognitive impairment
MIC: Microglia
MMSE: Mini-Mental State Examination
NFT: Neurofibrillary tangles
ODC: Oligodendrocytes
OPC: Oligodendrocyte precursors
ROSMAP: Religious Order Study/Memory and Aging Project
SNP: Single nucleotide polymorphism
SnATAC: Single-nuclei assay for transposase activity
SnRNA: Single-nuclei RNA-sequencing
