# Supplementary material for: Multi-omic derived cell-type specific Alzheimer disease polygenic risk scores
Source: Neurobiol Aging. Author manuscript; Available in PMC 2026 Apr 14. (PMC13077728; doi:10.1016/j.neurobiolaging.2025.07.009)

**Supplementary Materials**

**Supplementary Table 1**: ROSMAP participant characteristics by cognitive status.

|  | | AD cases | MCI cases | Controls |
| --- | --- | --- | --- | --- |
| Number of participants | | 407 | 258 | 351 |
| Mean Age, Years | | 90.8 | 89.4 | 87 |
| % Female | | 69.3 | 62.4 | 65.0 |
| Ancestry | European | 407 | 257 | 351 |
|  | African American | 0 | 0 | 0 |
|  | Hispanic | 0 | 1 | 0 |
| % APOE ε2 Carriers | | 11.4 | 11.7 | 20.6 |
| % APOE ε4 Carriers | | 38.1 | 23.4 | 16.3 |
| Median MMSE | | 13.5 | 26 | 28 |

**Supplementary Table 2**: Number of AD cases and controls in the Alzheimer Disease Genetics Consortium GWAS datasets.

| Cohort | Total | Cases | Controls |
| --- | --- | --- | --- |
| ACT1 | 2095 | 524 | 1571 |
| ACT3 | 1147 | 135 | 1012 |
| ADC1 | 2016 | 1508 | 508 |
| ADC2 | 771 | 615 | 156 |
| ADC3 | 1299 | 731 | 568 |
| ADC4 | 680 | 303 | 377 |
| ADC5 | 790 | 285 | 505 |
| ADC6 | 551 | 213 | 338 |
| ADC7 | 1304 | 514 | 790 |
| ADC8 | 902 | 421 | 481 |
| ADC9to12 | 3245 | 1479 | 1766 |
| ADNI | 441 | 268 | 173 |
| BIOCARD | 118 | 6 | 112 |
| CHAP2 | 171 | 27 | 144 |
| EAS | 150 | 9 | 141 |
| GSK | 1360 | 648 | 712 |
| LOAD | 3198 | 1738 | 1460 |
| MAYO | 1704 | 658 | 1046 |
| MIRAGE | 1207 | 481 | 726 |
| NBB | 128 | 80 | 48 |
| OHSU | 274 | 125 | 149 |
| PFIZER | 1457 | 695 | 762 |
| RMayo | 246 | 13 | 233 |
| TARCC1 | 504 | 323 | 181 |
| TARCC3 | 436 | 241 | 195 |
| TGEN2 | 982 | 617 | 365 |
| UKS | 766 | 596 | 170 |
| UMVUMSSM | 2253 | 1134 | 1119 |
| UMVUTARC2 | 441 | 253 | 188 |
| UPITT | 1990 | 1161 | 829 |
| WASHU1 | 505 | 318 | 187 |
| WASHU2 | 109 | 38 | 71 |
| WHICAP | 633 | 73 | 560 |
| Total | 33873 | 16230 | 17643 |

**Supplementary Table 3**: Cell count and percentage for each primary cell type in the snRNA and snATAC reference data.

| **Cell Type** | **AST** | **END** | **GAB** | **MIC** | **ODC** | **OPC** | **GLU** |
| --- | --- | --- | --- | --- | --- | --- | --- |
| snRNA | 13,390 | 1,407 | 16,207 | 4,907 | 19,908 | 4,216 | 43,963 |
| snRNA % | 12.88 | 1.35 | 15.58 | 4.72 | 19.14 | 4.05 | 42.27 |
| snATAC | 22,532 | 1,989 | 21,942 | 8,494 | 40,418 | 7,784 | 52,626 |
| snATAC % | 14.46 | 1.28 | 14.08 | 5.45 | 25.94 | 5.00 | 33.78 |

**Supplementary Table 4**: Number of SNPs included in the calculation of the ct-ADPRS for each neuronal subtype.

| **Neuronal Subtype** | **# SNPs** |
| --- | --- |
| GAB_LAMP5 | 2,111 |
| GAB_PVALB | 2,115 |
| GAB_SST | 1,954 |
| GAB_VIP | 1,908 |
| GLU_LAMP5 | 1,726 |
| GLU_RORB | 1,937 |
| GLU_SEMA3E | 2,059 |
| GLU_THEMIS | 2,008 |

**Supplementary Table 5**: Model results for snATAC-derived ct-ADPRS association with AD endophenotypes. Represents data shown in Supplementary Figure 2B. “N” represents the number of samples included in the single comparison. ‘Genome represents an ADPRS generated using all AD SNPs.

| **Cell Type** | **Variable** | **P-value** | **FDR** | **Estimate** | **Error** | **N** |
| --- | --- | --- | --- | --- | --- | --- |
| GENOME | Path. Diag. | 2E-06 | 1.17E-05 | 0.37 | 0.077 | 1111 |
| AST | Path. Diag. | 0.0003 | 0.0018 | 0.25 | 0.069 | 1111 |
| MIC | Path. Diag. | 0.0058 | 0.035 | 0.19 | 0.069 | 1111 |
| ODC | Path. Diag. | 0.17 | 1 | 0.094 | 0.068 | 1111 |
| OPC | Path. Diag. | 0.0017 | 0.010 | 0.21 | 0.068 | 1111 |
| GLU | Path. Diag. | 0.12 | 0.70 | 0.11 | 0.068 | 1111 |
| GAB | Path. Diag. | 0.99 | 1 | -0.00055 | 0.067 | 1111 |
| GENOME | AD vs. Control | 8.5E-06 | 5.08E-05 | 0.42 | 0.094 | 758 |
| AST | AD vs. Control | 0.046 | 0.28 | 0.17 | 0.084 | 758 |
| MIC | AD vs. Control | 0.22 | 1 | 0.099 | 0.081 | 758 |
| ODC | AD vs. Control | 0.99 | 1 | 0.00059 | 0.082 | 758 |
| OPC | AD vs. Control | 0.1 | 0.63 | 0.13 | 0.081 | 758 |
| GLU | AD vs. Control | 0.26 | 1 | 0.094 | 0.083 | 758 |
| GAB | AD vs. Control | 0.83 | 1 | 0.018 | 0.083 | 758 |
| GENOME | MCI vs. Control | 0.081 | 0.49 | 0.17 | 0.097 | 609 |
| AST | MCI vs. Control | 0.066 | 0.40 | 0.17 | 0.092 | 609 |
| MIC | MCI vs. Control | 0.72 | 1 | -0.031 | 0.086 | 609 |
| ODC | MCI vs. Control | 0.8 | 1 | -0.022 | 0.089 | 609 |
| OPC | MCI vs. Control | 0.28 | 1 | 0.093 | 0.087 | 609 |
| GLU | MCI vs. Control | 0.033 | 0.20 | 0.19 | 0.090 | 609 |
| GAB | MCI vs. Control | 0.75 | 1 | 0.029 | 0.091 | 609 |
| GENOME | AD vs. MCI | 0.0027 | 0.016 | 0.29 | 0.097 | 665 |
| AST | AD vs. MCI | 0.76 | 1 | -0.024 | 0.079 | 665 |
| MIC | AD vs. MCI | 0.041 | 0.25 | 0.18 | 0.086 | 665 |
| ODC | AD vs. MCI | 0.96 | 1 | 0.0038 | 0.080 | 665 |
| OPC | AD vs. MCI | 0.56 | 1 | 0.049 | 0.084 | 665 |
| GLU | AD vs. MCI | 0.84 | 1 | -0.016 | 0.081 | 665 |
| GAB | AD vs. MCI | 0.98 | 1 | 0.002 | 0.080 | 665 |
| GENOME | MMSE | 2.6E-07 | 1.56E-06 | -1.51 | 0.29 | 1110 |
| AST | MMSE | 0.0019 | 0.011 | -0.82 | 0.26 | 1110 |
| MIC | MMSE | 0.031 | 0.19 | -0.57 | 0.27 | 1110 |
| ODC | MMSE | 0.54 | 1 | -0.16 | 0.27 | 1110 |
| OPC | MMSE | 0.43 | 1 | -0.21 | 0.26 | 1110 |
| GLU | MMSE | 0.0073 | 0.044 | -0.72 | 0.27 | 1110 |
| GAB | MMSE | 0.71 | 1 | -0.10 | 0.27 | 1110 |
| GENOME | Aβ Burden | 0.0023 | 0.014 | 0.41 | 0.13 | 1103 |
| AST | Aβ Burden | 0.12 | 0.74 | 0.19 | 0.12 | 1103 |
| MIC | Aβ Burden | 0.062 | 0.37 | 0.23 | 0.12 | 1103 |
| ODC | Aβ Burden | 0.014 | 0.086 | 0.30 | 0.12 | 1103 |
| OPC | Aβ Burden | 0.074 | 0.45 | 0.21 | 0.12 | 1103 |
| GLU | Aβ Burden | 0.4 | 1 | 0.10 | 0.12 | 1103 |
| GAB | Aβ Burden | 0.092 | 0.55 | -0.20 | 0.12 | 1103 |
| GENOME | Diffuse Plaq | 0.18 | 1 | 0.036 | 0.027 | 1111 |
| AST | Diffuse Plaq | 0.088 | 0.53 | 0.041 | 0.024 | 1111 |
| MIC | Diffuse Plaq | 0.21 | 1 | 0.03 | 0.024 | 1111 |
| ODC | Diffuse Plaq | 0.66 | 1 | 0.011 | 0.024 | 1111 |
| OPC | Diffuse Plaq | 0.038 | 0.23 | 0.05 | 0.024 | 1111 |
| GLU | Diffuse Plaq | 1 | 1 | 0.00014 | 0.024 | 1111 |
| GAB | Diffuse Plaq | 0.33 | 1 | -0.024 | 0.024 | 1111 |
| GENOME | Neuritic Plaq | 0.00022 | 0.0013 | 0.095 | 0.026 | 1111 |
| AST | Neuritic Plaq | 0.0034 | 0.020 | 0.068 | 0.023 | 1111 |
| MIC | Neuritic Plaq | 0.016 | 0.098 | 0.056 | 0.023 | 1111 |
| ODC | Neuritic Plaq | 0.31 | 1 | 0.024 | 0.023 | 1111 |
| OPC | Neuritic Plaq | 0.031 | 0.19 | 0.050 | 0.023 | 1111 |
| GLU | Neuritic Plaq | 0.53 | 1 | 0.015 | 0.023 | 1111 |
| GAB | Neuritic Plaq | 0.93 | 1 | 0.002 | 0.023 | 1111 |
| GENOME | Tau Tangles | 7.3E-08 | 4.36E-07 | 1.32 | 0.24 | 1099 |
| AST | Tau Tangles | 0.00021 | 0.0013 | 0.82 | 0.22 | 1099 |
| MIC | Tau Tangles | 0.00079 | 0.0047 | 0.75 | 0.22 | 1099 |
| ODC | Tau Tangles | 0.073 | 0.44 | 0.40 | 0.22 | 1099 |
| OPC | Tau Tangles | 0.027 | 0.16 | 0.49 | 0.22 | 1099 |
| GLU | Tau Tangles | 0.5 | 1 | 0.15 | 0.22 | 1099 |
| GAB | Tau Tangles | 0.53 | 1 | 0.14 | 0.22 | 1099 |
| GENOME | NFT | 6.7E-06 | 4.02E-05 | 0.11 | 0.024 | 1111 |
| AST | NFT | 0.0013 | 0.0078 | 0.071 | 0.022 | 1111 |
| MIC | NFT | 0.012 | 0.075 | 0.056 | 0.022 | 1111 |
| ODC | NFT | 0.013 | 0.076 | 0.056 | 0.022 | 1111 |
| OPC | NFT | 0.012 | 0.073 | 0.055 | 0.022 | 1111 |
| GLU | NFT | 0.32 | 1 | 0.022 | 0.022 | 1111 |
| GAB | NFT | 0.9 | 1 | 0.0028 | 0.022 | 1111 |

**Supplementary Table 6**: Model results for snRNA-derived ct-ADPRS association with AD endophenotypes. Represents data shown in Supplementary Figure 2A. “N” represents the number of samples included in the single comparison. ‘Genome’ represents an ADPRS generated using all AD SNPs.

| **Cell Type** | **Variable** | **P-value** | **FDR** | **Estimate** | **Error** | **N** |
| --- | --- | --- | --- | --- | --- | --- |
| GENOME | Path. Diag. | 2E-06 | 1.17E-05 | 0.37 | 0.077 | 1111 |
| AST | Path. Diag. | 5.5E-05 | 0.00033 | 0.28 | 0.071 | 1111 |
| MIC | Path. Diag. | 0.0089 | 0.054 | 0.18 | 0.068 | 1111 |
| ODC | Path. Diag. | 0.03 | 0.18 | 0.15 | 0.069 | 1111 |
| OPC | Path. Diag. | 0.18 | 1 | 0.092 | 0.068 | 1111 |
| GLU | Path. Diag. | 0.0013 | 0.0080 | 0.22 | 0.069 | 1111 |
| GAB | Path. Diag. | 0.085 | 0.51 | 0.12 | 0.069 | 1111 |
| GENOME | AD vs. Control | 8.5E-06 | 5.08E-05 | 0.42 | 0.094 | 758 |
| AST | AD vs. Control | 0.073 | 0.44 | 0.15 | 0.086 | 758 |
| MIC | AD vs. Control | 0.26 | 1 | 0.093 | 0.083 | 758 |
| ODC | AD vs. Control | 0.012 | 0.070 | 0.21 | 0.085 | 758 |
| OPC | AD vs. Control | 0.0025 | 0.015 | 0.26 | 0.085 | 758 |
| GLU | AD vs. Control | 0.052 | 0.31 | 0.16 | 0.083 | 758 |
| GAB | AD vs. Control | 0.011 | 0.063 | 0.22 | 0.084 | 758 |
| GENOME | MCI vs. Control | 0.081 | 0.49 | 0.17 | 0.097 | 609 |
| AST | MCI vs. Control | 0.56 | 1 | -0.051 | 0.087 | 609 |
| MIC | MCI vs. Control | 0.96 | 1 | -0.0042 | 0.084 | 609 |
| ODC | MCI vs. Control | 0.31 | 1 | 0.087 | 0.087 | 609 |
| OPC | MCI vs. Control | 0.13 | 0.76 | 0.14 | 0.089 | 609 |
| GLU | MCI vs. Control | 0.52 | 1 | 0.058 | 0.091 | 609 |
| GAB | MCI vs. Control | 0.29 | 1 | 0.096 | 0.091 | 609 |
| GENOME | AD vs. MCI | 0.0027 | 0.016 | 0.29 | 0.097 | 665 |
| AST | AD vs. MCI | 0.033 | 0.20 | 0.18 | 0.085 | 665 |
| MIC | AD vs. MCI | 0.098 | 0.59 | 0.14 | 0.085 | 665 |
| ODC | AD vs. MCI | 0.37 | 1 | 0.079 | 0.087 | 665 |
| OPC | AD vs. MCI | 0.11 | 0.64 | 0.14 | 0.086 | 665 |
| GLU | AD vs. MCI | 0.16 | 0.95 | 0.12 | 0.087 | 665 |
| GAB | AD vs. MCI | 0.26 | 1 | 0.093 | 0.083 | 665 |
| GENOME | MMSE | 2.6E-07 | 1.56E-06 | -1.51 | 0.29 | 1110 |
| AST | MMSE | 0.11 | 0.65 | -0.43 | 0.27 | 1110 |
| MIC | MMSE | 0.0039 | 0.024 | -0.77 | 0.27 | 1110 |
| ODC | MMSE | 0.035 | 0.21 | -0.57 | 0.27 | 1110 |
| OPC | MMSE | 0.058 | 0.35 | -0.51 | 0.27 | 1110 |
| GLU | MMSE | 0.055 | 0.33 | -0.52 | 0.27 | 1110 |
| GAB | MMSE | 0.14 | 0.81 | -0.41 | 0.27 | 1110 |
| GENOME | Aβ Burden | 0.0023 | 0.014 | 0.41 | 0.13 | 1103 |
| AST | Aβ Burden | 0.0069 | 0.042 | 0.33 | 0.12 | 1103 |
| MIC | Aβ Burden | 0.12 | 0.74 | 0.19 | 0.12 | 1103 |
| ODC | Aβ Burden | 0.35 | 1 | 0.11 | 0.12 | 1103 |
| OPC | Aβ Burden | 0.33 | 1 | 0.12 | 0.12 | 1103 |
| GLU | Aβ Burden | 0.058 | 0.35 | 0.23 | 0.12 | 1103 |
| GAB | Aβ Burden | 0.088 | 0.53 | 0.21 | 0.12 | 1103 |
| GENOME | Diffuse Plaq | 0.18 | 1 | 0.036 | 0.027 | 1111 |
| AST | Diffuse Plaq | 0.18 | 1 | 0.033 | 0.025 | 1111 |
| MIC | Diffuse Plaq | 0.12 | 0.74 | 0.038 | 0.024 | 1111 |
| ODC | Diffuse Plaq | 0.73 | 1 | 0.0085 | 0.025 | 1111 |
| OPC | Diffuse Plaq | 0.97 | 1 | -0.00086 | 0.025 | 1111 |
| GLU | Diffuse Plaq | 0.19 | 1 | 0.033 | 0.025 | 1111 |
| GAB | Diffuse Plaq | 0.33 | 1 | 0.024 | 0.025 | 1111 |
| GENOME | Neuritic Plaq | 0.00022 | 0.0013 | 0.095 | 0.026 | 1111 |
| AST | Neuritic Plaq | 0.0033 | 0.020 | 0.070 | 0.024 | 1111 |
| MIC | Neuritic Plaq | 0.077 | 0.46 | 0.041 | 0.023 | 1111 |
| ODC | Neuritic Plaq | 0.19 | 1 | 0.031 | 0.024 | 1111 |
| OPC | Neuritic Plaq | 0.86 | 1 | 0.0043 | 0.024 | 1111 |
| GLU | Neuritic Plaq | 0.022 | 0.13 | 0.055 | 0.024 | 1111 |
| GAB | Neuritic Plaq | 0.063 | 0.38 | 0.044 | 0.024 | 1111 |
| GENOME | Tau Tangles | 7.3E-08 | 4.36E-07 | 1.32 | 0.24 | 1099 |
| AST | Tau Tangles | 0.0075 | 0.045 | 0.60 | 0.23 | 1099 |
| MIC | Tau Tangles | 0.0047 | 0.028 | 0.63 | 0.22 | 1099 |
| ODC | Tau Tangles | 0.0015 | 0.0089 | 0.72 | 0.23 | 1099 |
| OPC | Tau Tangles | 0.02 | 0.12 | 0.53 | 0.23 | 1099 |
| GLU | Tau Tangles | 0.28 | 1 | 0.24 | 0.23 | 1099 |
| GAB | Tau Tangles | 0.038 | 0.23 | 0.47 | 0.23 | 1099 |
| GENOME | NFT | 6.7E-06 | 4.02E-05 | 0.11 | 0.024 | 1111 |
| AST | NFT | 0.0098 | 0.059 | 0.058 | 0.023 | 1111 |
| MIC | NFT | 0.0027 | 0.016 | 0.066 | 0.022 | 1111 |
| ODC | NFT | 0.0042 | 0.025 | 0.064 | 0.022 | 1111 |
| OPC | NFT | 0.11 | 0.69 | 0.036 | 0.023 | 1111 |
| GLU | NFT | 0.32 | 1 | 0.023 | 0.023 | 1111 |
| GAB | NFT | 0.22 | 1 | 0.028 | 0.023 | 1111 |

**Supplementary Table 7**: Model results for multi-omic neuron subtype ADPRS association with AD endophenotypes. Represents data shown in Supplementary Figure 3. “N” represents the number of samples included in the single comparison.

| **Cell Type** | **Variable** | **P-value** | **FDR** | **Estimate** | **Error** | **N** |
| --- | --- | --- | --- | --- | --- | --- |
| GLU_THEMIS | Path. Diag. | 0.43 | 1 | -0.053 | 0.068 | 1111 |
| GLU_SEMA3E | Path. Diag. | 0.8 | 1 | 0.017 | 0.067 | 1111 |
| GLU_RORB | Path. Diag. | 0.8 | 1 | -0.017 | 0.067 | 1111 |
| GLU_LAMP5 | Path. Diag. | 0.66 | 1 | 0.029 | 0.067 | 1111 |
| GAB_PVALB | Path. Diag. | 0.3 | 1 | 0.070 | 0.067 | 1111 |
| GAB_SST | Path. Diag. | 0.0089 | 0.072 | 0.18 | 0.068 | 1111 |
| GAB_VIP | Path. Diag. | 0.67 | 1 | 0.029 | 0.068 | 1111 |
| GAB_LAMP5 | Path. Diag. | 0.15 | 1 | 0.098 | 0.068 | 1111 |
| GLU_THEMIS | AD v Control | 0.36 | 1 | -0.074 | 0.081 | 758 |
| GLU_SEMA3E | AD v Control | 0.23 | 1 | 0.098 | 0.082 | 758 |
| GLU_RORB | AD v Control | 0.45 | 1 | -0.060 | 0.079 | 758 |
| GLU_LAMP5 | AD v Control | 0.19 | 1 | -0.11 | 0.083 | 758 |
| GAB_PVALB | AD v Control | 0.92 | 1 | -0.0080 | 0.081 | 758 |
| GAB_SST | AD v Control | 0.18 | 1 | 0.11 | 0.081 | 758 |
| GAB_VIP | AD v Control | 0.062 | 0.50 | -0.16 | 0.084 | 758 |
| GAB_LAMP5 | AD v Control | 0.49 | 1 | -0.058 | 0.084 | 758 |
| GLU_THEMIS | MCI v Control | 0.88 | 1 | -0.013 | 0.087 | 609 |
| GLU_SEMA3E | MCI v Control | 0.042 | 0.34 | 0.17 | 0.084 | 609 |
| GLU_RORB | MCI v Control | 0.74 | 1 | -0.027 | 0.083 | 609 |
| GLU_LAMP5 | MCI v Control | 0.31 | 1 | -0.089 | 0.088 | 609 |
| GAB_PVALB | MCI v Control | 0.68 | 1 | -0.035 | 0.085 | 609 |
| GAB_SST | MCI v Control | 0.97 | 1 | 0.0034 | 0.084 | 609 |
| GAB_VIP | MCI v Control | 0.31 | 1 | -0.092 | 0.090 | 609 |
| GAB_LAMP5 | MCI v Control | 0.69 | 1 | 0.034 | 0.086 | 609 |
| GLU_THEMIS | AD v MCI | 0.52 | 1 | -0.055 | 0.085 | 665 |
| GLU_SEMA3E | AD v MCI | 0.6 | 1 | -0.045 | 0.085 | 665 |
| GLU_RORB | AD v MCI | 0.95 | 1 | 0.0054 | 0.085 | 665 |
| GLU_LAMP5 | AD v MCI | 0.81 | 1 | 0.020 | 0.083 | 665 |
| GAB_PVALB | AD v MCI | 0.86 | 1 | -0.015 | 0.084 | 665 |
| GAB_SST | AD v MCI | 0.27 | 1 | 0.097 | 0.087 | 665 |
| GAB_VIP | AD v MCI | 0.37 | 1 | -0.071 | 0.080 | 665 |
| GAB_LAMP5 | AD v MCI | 0.16 | 1 | -0.11 | 0.081 | 665 |
| GLU_THEMIS | MMSE | 0.89 | 1 | 0.036 | 0.27 | 1110 |
| GLU_SEMA3E | MMSE | 0.67 | 1 | 0.11 | 0.27 | 1110 |
| GLU_RORB | MMSE | 0.76 | 1 | -0.081 | 0.26 | 1110 |
| GLU_LAMP5 | MMSE | 0.94 | 1 | -0.019 | 0.27 | 1110 |
| GAB_PVALB | MMSE | 0.9 | 1 | -0.034 | 0.27 | 1110 |
| GAB_SST | MMSE | 0.033 | 0.26 | -0.57 | 0.26 | 1110 |
| GAB_VIP | MMSE | 0.53 | 1 | 0.17 | 0.27 | 1110 |
| GAB_LAMP5 | MMSE | 0.47 | 1 | 0.19 | 0.27 | 1110 |
| GLU_THEMIS | Aβ Burden | 0.24 | 1 | 0.14 | 0.12 | 1103 |
| GLU_SEMA3E | Aβ Burden | 0.58 | 1 | -0.068 | 0.12 | 1103 |
| GLU_RORB | Aβ Burden | 0.62 | 1 | -0.060 | 0.12 | 1103 |
| GLU_LAMP5 | Aβ Burden | 0.8 | 1 | -0.031 | 0.12 | 1103 |
| GAB_PVALB | Aβ Burden | 0.74 | 1 | -0.040 | 0.12 | 1103 |
| GAB_SST | Aβ Burden | 0.0037 | 0.030 | 0.35 | 0.12 | 1103 |
| GAB_VIP | Aβ Burden | 0.26 | 1 | -0.14 | 0.12 | 1103 |
| GAB_LAMP5 | Aβ Burden | 0.68 | 1 | -0.050 | 0.12 | 1103 |
| GLU_THEMIS | Diffuse Plaq | 0.26 | 1 | -0.027 | 0.024 | 1111 |
| GLU_SEMA3E | Diffuse Plaq | 0.014 | 0.11 | -0.060 | 0.024 | 1111 |
| GLU_RORB | Diffuse Plaq | 0.69 | 1 | -0.0097 | 0.024 | 1111 |
| GLU_LAMP5 | Diffuse Plaq | 0.64 | 1 | 0.011 | 0.024 | 1111 |
| GAB_PVALB | Diffuse Plaq | 0.82 | 1 | 0.0055 | 0.024 | 1111 |
| GAB_SST | Diffuse Plaq | 0.09 | 0.72 | 0.041 | 0.024 | 1111 |
| GAB_VIP | Diffuse Plaq | 0.7 | 1 | -0.0093 | 0.024 | 1111 |
| GAB_LAMP5 | Diffuse Plaq | 0.39 | 1 | -0.021 | 0.024 | 1111 |
| GLU_THEMIS | Neuritic Plaq | 0.83 | 1 | -0.0049 | 0.023 | 1111 |
| GLU_SEMA3E | Neuritic Plaq | 0.93 | 1 | -0.0021 | 0.023 | 1111 |
| GLU_RORB | Neuritic Plaq | 0.79 | 1 | 0.0063 | 0.023 | 1111 |
| GLU_LAMP5 | Neuritic Plaq | 0.045 | 0.36 | 0.047 | 0.023 | 1111 |
| GAB_PVALB | Neuritic Plaq | 0.68 | 1 | 0.0097 | 0.023 | 1111 |
| GAB_SST | Neuritic Plaq | 0.00018 | 0.0014 | 0.087 | 0.023 | 1111 |
| GAB_VIP | Neuritic Plaq | 0.62 | 1 | -0.012 | 0.023 | 1111 |
| GAB_LAMP5 | Neuritic Plaq | 0.39 | 1 | 0.020 | 0.023 | 1111 |
| GLU_THEMIS | Tau Tangles | 0.054 | 0.43 | -0.43 | 0.22 | 1099 |
| GLU_SEMA3E | Tau Tangles | 0.75 | 1 | -0.07 | 0.22 | 1099 |
| GLU_RORB | Tau Tangles | 0.77 | 1 | -0.066 | 0.22 | 1099 |
| GLU_LAMP5 | Tau Tangles | 0.54 | 1 | 0.14 | 0.22 | 1099 |
| GAB_PVALB | Tau Tangles | 0.4 | 1 | -0.19 | 0.22 | 1099 |
| GAB_SST | Tau Tangles | 0.74 | 1 | 0.073 | 0.22 | 1099 |
| GAB_VIP | Tau Tangles | 0.97 | 1 | -0.0076 | 0.22 | 1099 |
| GAB_LAMP5 | Tau Tangles | 0.95 | 1 | 0.013 | 0.22 | 1099 |
| GLU_THEMIS | NFT | 0.66 | 1 | -0.0097 | 0.022 | 1111 |
| GLU_SEMA3E | NFT | 0.77 | 1 | -0.0064 | 0.022 | 1111 |
| GLU_RORB | NFT | 0.83 | 1 | 0.0047 | 0.022 | 1111 |
| GLU_LAMP5 | NFT | 0.043 | 0.34 | 0.045 | 0.022 | 1111 |
| GAB_PVALB | NFT | 0.79 | 1 | 0.0058 | 0.022 | 1111 |
| GAB_SST | NFT | 0.13 | 1 | 0.033 | 0.022 | 1111 |
| GAB_VIP | NFT | 0.64 | 1 | 0.010 | 0.022 | 1111 |
| GAB_LAMP5 | NFT | 0.74 | 1 | 0.0073 | 0.022 | 1111 |

**Supplementary Table 8**: Association of variants included in the SST+ GABAergic neuron ct-ADPRS with neuritic plaque burden, amyloid β, and AD status.

See attached “ctPRS Supplementary tables 8-10.xlsx”

**Supplementary Table 9**: Association of variants within 100 kb of *NPY* variant rs3940268 with neuritic plaque burden, amyloid β, and AD status.

See attached “ctPRS Supplementary tables 8-10.xlsx”

**Supplementary Table 10**: Association of variants within 100 kb of *GHR* variant rs10941583 with neuritic plaque burden, amyloid β, and AD status.

See attached “ctPRS Supplementary tables 8-10.xlsx”

**Supplementary Figure 1**: Distributions of cell type specificity scores for peaks and genes for only primary cell types (**A-B**). Black dots represent the threshold for selecting the top 10% most cell type specific peaks and genes. Specificity thresholds for peaks and genes in the primary cell type analyses are plotted against each other in **C**.

**A. B.**


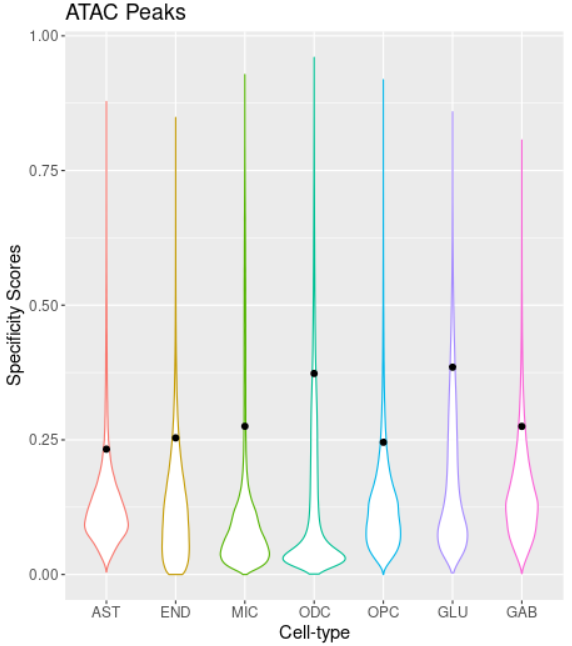

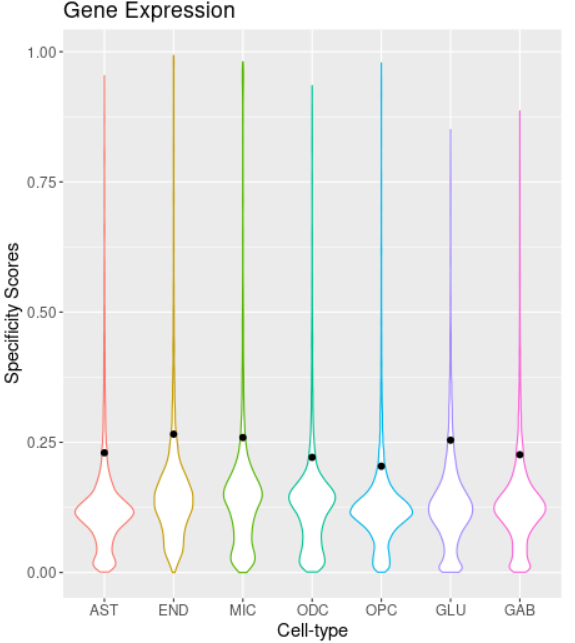


**C.**


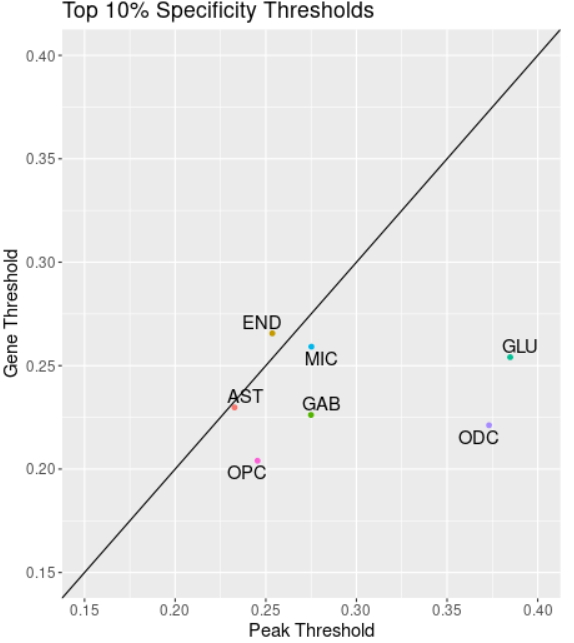


**Supplementary Figure 2**: UpSetR plots displaying the number of SNPs specific to and shared between each of the six cell types considered for each ct-ADPRS range, (**A**) snRNA cell type specific genes and (**B**) snATAC cell type specific peaks. Rows show the number of SNPs included for each cell type, with the total shown on a bar-chart on the right. Individual black circles represent sets of SNPs specific to a single cell type, and black circles connected by lines represent sets of SNPs shared between two or more cell types. The total number of SNPs for each intersection group is displayed in a bar-chart above.


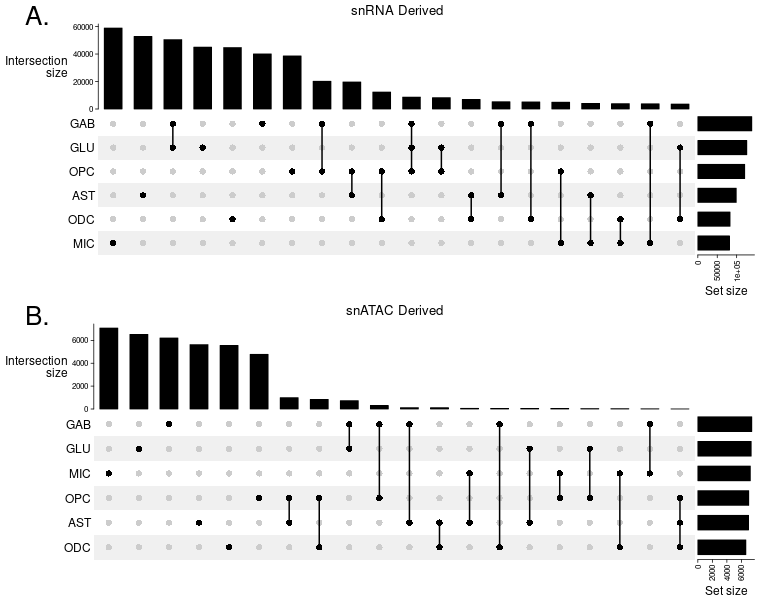


**Supplementary Figure 3**: Correlation plots of ct-ADPRS and overall ADPRSs for neuron subtypes. Blue circles represent correlation ‘r’ values between two cell types’ ADPRSs. The first row and column of each correlation plot is the overall ‘Genome’ ADPRS generated by using all SNPs, regardless of cell type specificity.


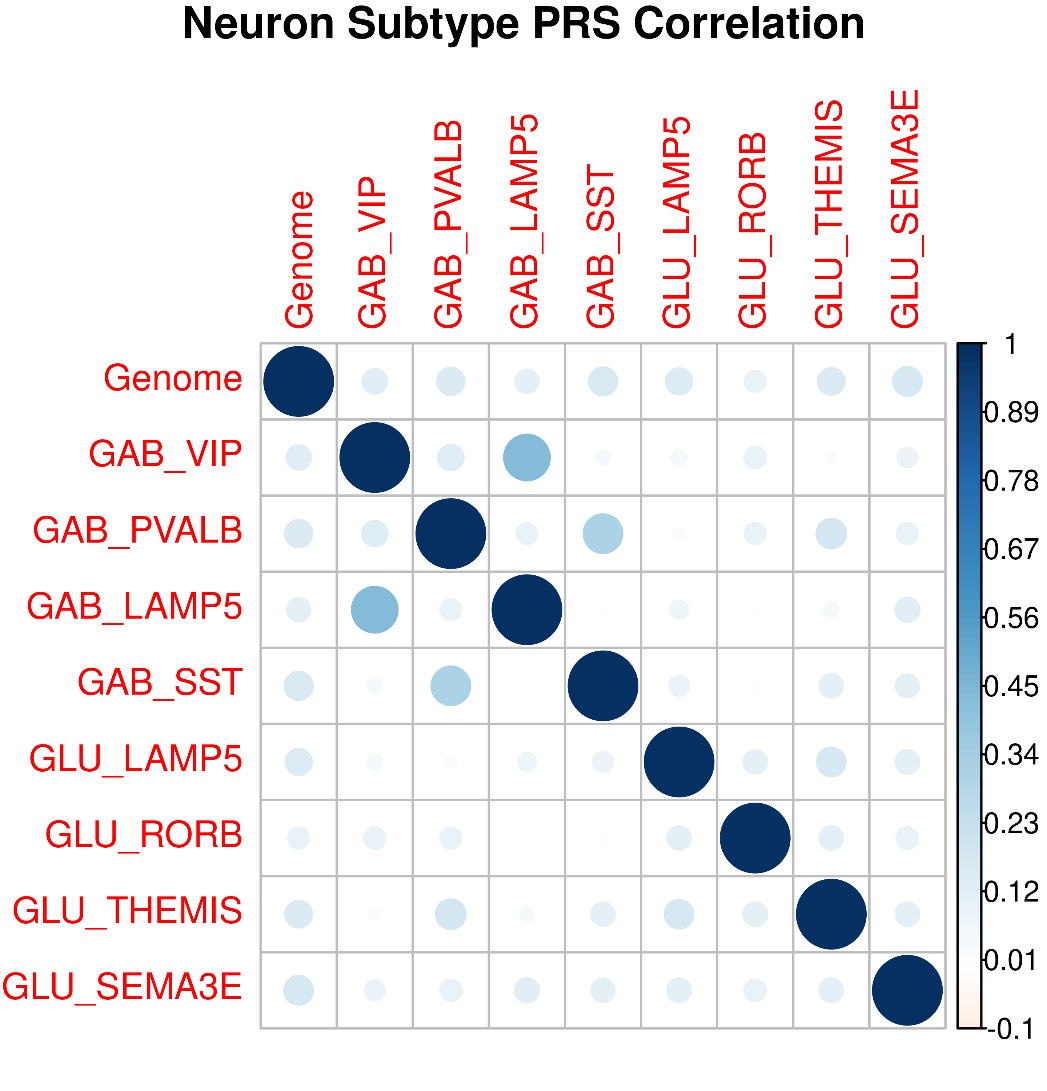


**Supplementary Figure 4**: Extension of Figure 3; ct-ADPRS association with AD endophenotypes**.** For each ct-ADPRS, variants on (**A**) snRNA cell type specific genes or (**B**) snATAC cell type specific peaks, we show logistic model results for pathological AD diagnosis (niareagan 0-1 vs 2-3) and cognitive AD diagnosis (CT = Not cognitively impaired, MCI = Mild cognitive impairment with no non-AD cause, AD = Alzheimer’s dementia with no other cause of dementia) and linear model results for MMSE score, Aβ burden quantified by immunohistochemistry, diffuse and neuritic plaque burden quantified by microscopy, immunohistochemistry-derived paired helical filament tau tangle density, and microscopy derived NFT burden. Models are adjusted for age, sex, APOE e2 and e4 dosage, and three genetic principal components. Cognitive diagnosis and MMSE models also include a covariate for years of education. Bars represent the -log10(FDR) for each of the six cell types considered as well as ‘Genome’ which represents an ADPRS generated using all AD SNPs. The dotted line represents a p-value of 0.05 while the black lines represent an FDR of 0.05. Effect size is shown above each bar for each significant ADPRS.


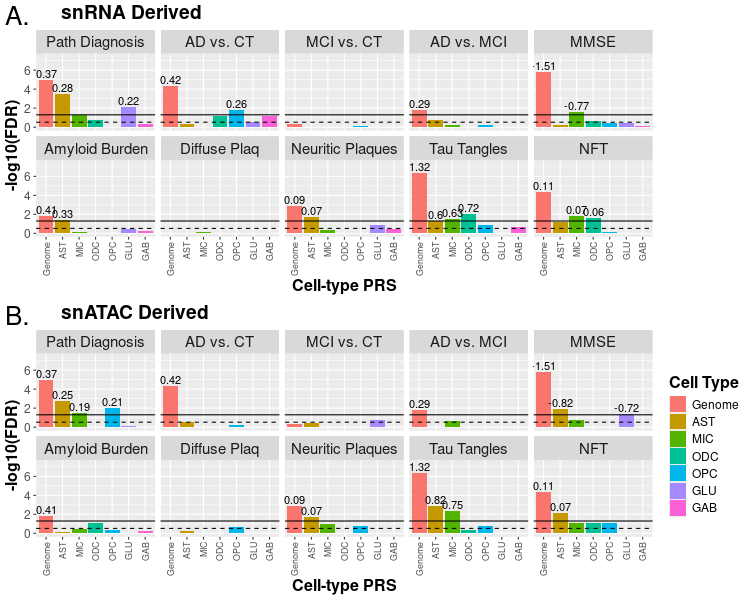


**Supplementary Figure 5**: Extension of Figure 4: multi-omic ct-ADPRS for neuron subtypes. We calculate a ct-ADPRS for each of eight neuron subtypes, four GABAergic and four glutamatergic. For each subtype we show logistic model results for pathological AD diagnosis (niareagan 0-1 vs 2-3) and cognitive AD diagnosis (CT = Not cognitively impaired, MCI = Mild cognitive impairment with no non-AD cause, AD = Alzheimer’s dementia with no other cause of dementia) and linear model results for MMSE score, Aβ burden quantified by immunohistochemistry, diffuse and neuritic plaque burden quantified by microscopy, immunohistochemistry-derived paired helical filament tau tangle density, and microscopy derived NFT burden. Models are adjusted for age, sex, APOE e2 and e4 dosage, and three genetic principal components. Cognitive diagnosis and MMSE models also include a covariate for years of education. Bars represent the -log10(FDR) for each of the eight neuron subtypes considered. The dotted line represents a p-value of 0.05 while the black lines represent an FDR of 0.05. Effect size is shown above each bar for each significant ADPRS.


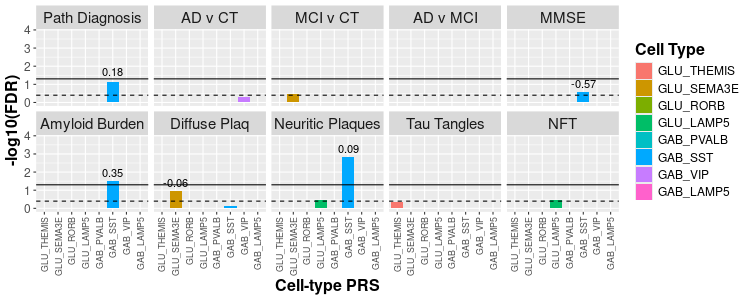


**Supplementary Figure 6**: Expression of NPY and GHR in brain cell types and neuron subtypes. Violin plots show the average normalized gene expression among cell types. Due to the low relative expression of NPY, dots are included to show individual cells’ expression.

**
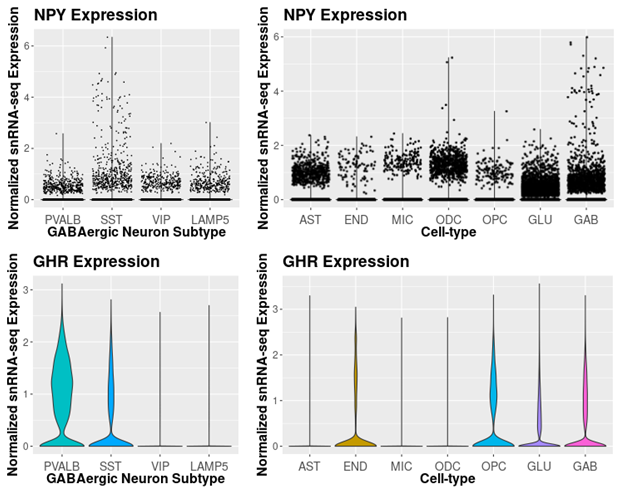
**

**Supplementary Figure 7**: UMAP plots showing the normalized expression of *NOS1*, *TACR1*, *NPY*, and *CHODL* in SST+ GABAergic neurons.


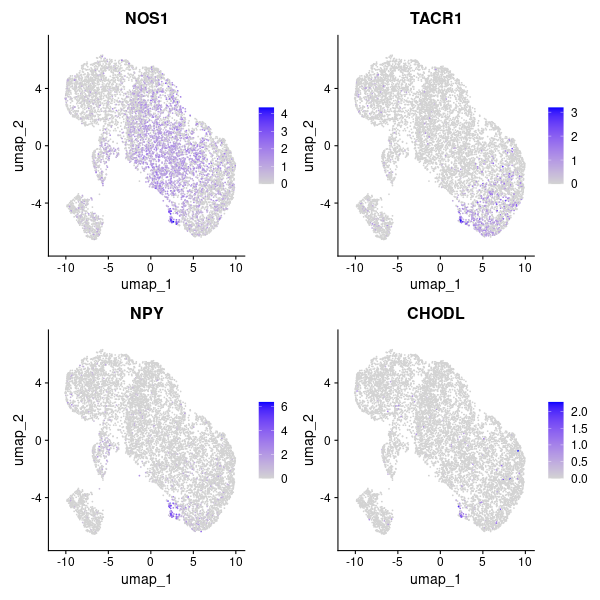


**Supplementary Figure 8**: Locus zoom plot showing the association between Amyloid β burden with variants near the *GHR* gene. The X-axis signifies the SNP position, and the significance of association is shown by the -log_10_(p-value) on the Y-axis.


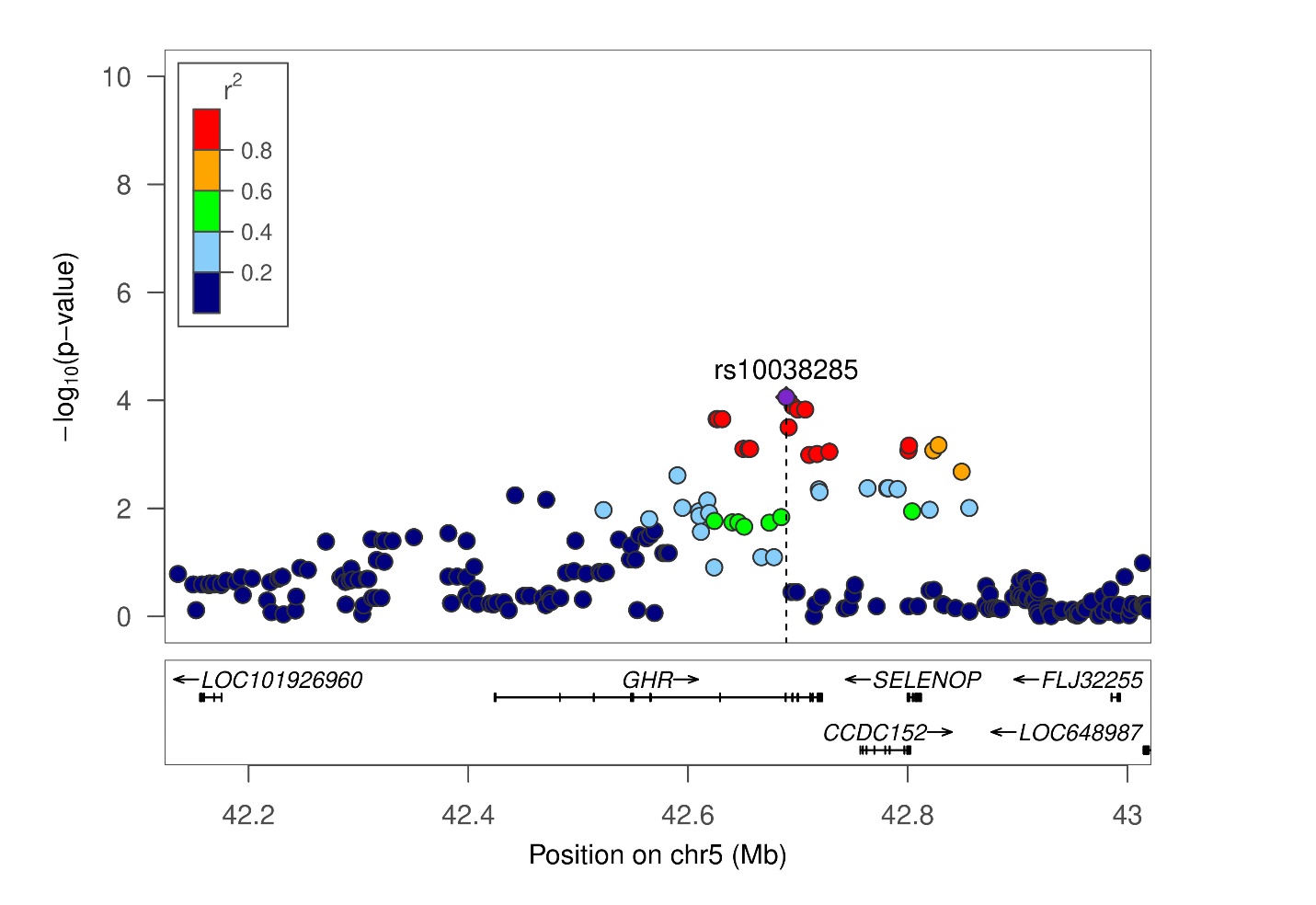

Supplement: Supplementary Tables of Cohorts [file NIHMS2155189-supplement-Supplementary_Tables_of_Cohorts.docx]
